# On the prediction of DNA-binding proteins only from primary sequences: A deep learning approach

**DOI:** 10.1371/journal.pone.0188129

**Published:** 2017-12-29

**Authors:** Yu-Hui Qu, Hua Yu, Xiu-Jun Gong, Jia-Hui Xu, Hong-Shun Lee

**Affiliations:** 1 School of Computer Science and Technology, Tianjin University, Nankai, Tianjin, China, 30072; 2 Tianjin Key Laboratory of Cognitive Computing and Application, Nankai, Tianjin, China, 30072; 3 Beijing KEDONG Electric Power Control System Co. LTD, Qinghe, Beijing, China, 100192; Harbin Institute of Technology Shenzhen Graduate School, CHINA

## Abstract

DNA-binding proteins play pivotal roles in alternative splicing, RNA editing, methylating and many other biological functions for both eukaryotic and prokaryotic proteomes. Predicting the functions of these proteins from primary amino acids sequences is becoming one of the major challenges in functional annotations of genomes. Traditional prediction methods often devote themselves to extracting physiochemical features from sequences but ignoring motif information and location information between motifs. Meanwhile, the small scale of data volumes and large noises in training data result in lower accuracy and reliability of predictions. In this paper, we propose a deep learning based method to identify DNA-binding proteins from primary sequences alone. It utilizes two stages of convolutional neutral network to detect the function domains of protein sequences, and the long short-term memory neural network to identify their long term dependencies, an binary cross entropy to evaluate the quality of the neural networks. When the proposed method is tested with a realistic DNA binding protein dataset, it achieves a prediction accuracy of 94.2% at the Matthew’s correlation coefficient of 0.961. Compared with the LibSVM on the arabidopsis and yeast datasets via independent tests, the accuracy raises by 9% and 4% respectively. Comparative experiments using different feature extraction methods show that our model performs similar accuracy with the best of others, but its values of sensitivity, specificity and AUC increase by 27.83%, 1.31% and 16.21% respectively. Those results suggest that our method is a promising tool for identifying DNA-binding proteins.

## Introduction

One vital function of proteins is DNA-binding that play pivotal roles in alternative splicing, RNA editing, methylating and many other biological functions for both eukaryotic and prokaryotic proteomes [1]. Currently, both computational and experimental techniques have been developed to identify the DNA binding proteins. Due to the pitfalls of time-consuming and expensive in experimental identifications, computational approaches are highly desired to distinguish the DNA-binding proteins from the explosively increased amount of newly discovered proteins. So far, numerous structure or sequence based predictors for determining DNA-binding proteins have been proposed [2–4]. Structure based predictions normally gain high accuracy on the basis of availability of many physiochemical characters. However, they are only applied to small number of proteins with high-resolution three-dimensional structures. Thus, uncovering DNA binding proteins from their primary sequences alone is becoming an urgent task in functional annotations of genomics with the availability of huge volumes of protein sequence data.

In the past decades, a series of computational methods for identifying of DNA-binding proteins using only primary sequences have been proposed. Among these methods, building a meaningful feature set and choosing an appropriate machine learning algorithm are two crucial steps to make the predictions successful [5]. Cai et al. first developed the SVM algorithm, SVM-Prot, in which the feature set came from three protein descriptors, composition (C), transition (T) and distribution (D)for extracting seven physiochemical characters of amino acids [2]. Kumar et al. trained a SVM model using amino acid composition and evolutionary information in the form of PSSM profiles [1]. iDNA-Prot used random forest algorithm as the predictor engine by incorporating the features into the general form of pseudo amino acid composition that were extracted from protein sequences via a “grey model” [3]. Zou et al. trained a SVM classifier, in which the feature set came from three different feature transformation methods of four kinds of protein properties [4]. Lou et al. proposed a prediction method of DNA-binding proteins by performing the feature rank using random forest and the wrapper-based feature selection using a forward best-first search strategy [6]. Ma et al. used the random forest classifier with a hybrid feature set by incorporating binding propensity of DNA-binding residues [7]. Professor Liu’s group developed several novel tools for predicting DNA-Binding proteins, such as iDNA-Prot|dis by incorporating amino acid distance-pairs and reducing alphabet profiles into the general pseudo amino acid composition [8], PseDNA-Pro by combining PseAAC and physiochemical distance transformations [9], iDNAPro-PseAAC by combining pseudo amino acid composition and profile-based protein representation [10], iDNA-KACC by combining auto-cross covariance transformation and ensemble learning [11]. Zhou et al. encoded a protein sequence at multi-scale by seven properties, including their qualitative and quantitative descriptions, of amino acids for predicting protein interactions [5]. Also there are several general purpose protein feature extraction tools such as Pse-in-One [12] and Pse-Analysis [13]. They generated feature vectors by a user-defined schema and make them more flexible.

Deep learning is now one of the most active fields in machine learning and has achieved big success in computer vision [14], speech recognition [15] and natural language processing [16]. It is composed of multiple linear and non-linear transformations to model high-level abstractions by using a deep graph with multiple processing layers. Convolutional neural networks (CNN) and Long short term memory neural networks(LSTM) are two typical architectures of deep learning. Communities from computation biology are making efforts into deep learning to solve their biological problems [17] ranged from DNA, RNA binding specifity prediction [18–20] to protein secondary structure [21], folding [22], and contact map [23] recognitions. Most of them make predictions using not only sequences, but additional information, such as transcription [18] and evolutionary profiles [21]. Few of them use sequences information alone. Furthermore, Asgari et al. have derived a continuous distributed representation of biological sequences to make the development rapidly [24].

Since deep learning techniques have been successful in other disciplines, we aim to investigate whether deep learning networks could achieve notable improvements in the field of identifying DNA binding proteins only using sequence information. In this work, we propose a deep learning based method to predict DNA-binding proteins from primary sequences. The model utilizes two stages of convolutional neutral network to detect the function domains of protein sequences, and the long short-term memory neural network to identify their long term dependence, an binary cross entropy to evaluate the quality of the neural networks. It overcomes more human intervention in feature selection procedure than in traditional machine learning methods, since all features are learned automatically. It uses filters to detect the function domains of a sequence. The domain position information are encoded by feature maps produced by the LSTM. Intensive experiments show its remarkable prediction power with high generality and reliability.

## Materials and methods

### Data sets

The raw protein sequences are extracted from the Swiss-Prot dataset, a manually annotated and reviewed subset of UniProt. It is a comprehensive, high-quality and freely accessible database of protein sequences and functional information. We collect 551, 193 proteins as the raw dataset from the release version 2016.5 of Swiss-Prot.

To obtain DNA-Binding proteins, we extract sequences from raw dataset by searching keyword “DNA-Binding”, then remove those sequences with length less than 40 or greater than 1,000 amino acids. Finally 42,257 protein sequences are selected as positive samples. We randomly select 42,310 non-DNA-Binding proteins as negative samples from the rest of the dataset by using the query condition “molecule function and length [40 to 1,000]”. For both of positive and negative samples, 80% of them are randomly selected as the training set, rest of them as the testing set. Also, to validate the generality of our model, two additional testing sets (Yeast and Arabidopsis) from literature [25] are used. See Table 1 for details.

**Table 1:** Equal data set.

Data set	DNA-binding	non-DNA-binding	Total
Original set	42,257	42,310	84,567
Train set	33,805	33,848	67,653
Test set	8,452	8,462	16,914
Yeast	100	100	200
Arabidopsis	100	100	200

In reality, the number of none-DNA-binding proteins is far greater than the one of DNA-binding proteins and the majority of DNA-binding protein data sets are imbalanced. Therefore we simulate a realistic data set by using the same positive samples in the equal set, and using the query conditions ‘molecule function and length [40 to 1,000]’ to construct negative samples from the dataset which doesn’t include those positive samples, see Table 2. The validation datasets were also obtained using the method in the literary [25], adding a condition ‘(sequence length ≤ 1000)’. Finally 104 sequences with DNA-binding and 480 sequences without DNA-binding were obtained.

**Table 2:** Realistic data set.

Data set	DNA-binding	non-DNA-binding	Total
Original set	42,257	341,481	383,738
Train set	33,805	273,185	306,990
Test set	8,452	68,296	76,748
Validation set	104	480	584

In order to further verify the generalization of the model, multi-species datasets including human, mouse and rice species are constructed using the method above. For the details, see Table 3.

**Table 3:** Multi-species data set.

Species	Data set	DNA-binding	non-DNA-binding	Total
Human	Original set	6,932	6,932	13,864
	Train set	5,546	5,546	11,092
	Test set	1,386	1,386	2,772
Mouse	Original set	4,883	4,883	9,766
	Train set	3,907	3,907	7,814
	Test set	976	976	1,952
Rice	Original set	4,501	4,501	9,002
	Train set	3,601	3,601	7,202
	Test set	900	900	1,800

For the traditional sequence-based classification methods, the redundancy of sequences in the training dataset often leads to over-fitting of the prediction model. Meanwhile, sequences in testing sets of Yeast and Arabidopsis may be included in the training dataset or share high similarity with some sequences in training dataset. These overlapped sequences might result in the pseudo performance in testing. Thus, we construct low-redundancy versions of both equal and realistic datasets to validate if our method works on such situations. We first remove the sequences in the datasets of Yeast and Arabidopsis. Then the CD-HIT tool with lowest threshold value 0.7 is applied to remove the sequence redundancy, see Table 4 for details of the datasets.

**Table 4:** Low-redundancy versions of the equal and realistic datasets.

Data set	DNA-binding	non-DNA-binding	Total
Equal dataset	17,327	26,443	43,770
Realistic dataset	17,327	125,792	143,119

### Methods

Just like the natural language in the real world, letters working together in different combinations construct words, words combining with each other in different ways form phrases. Processing words in a document can convey the topic of the document and its meaningful content. In this work, a protein sequence is analogous to a document, amino acid to word, and motif to phrase. Mining relationships among them would yield higher level information on the behavioral properties of the physical entities corresponding to the sequences.

#### Deep learning model structure

**Fig 1:**
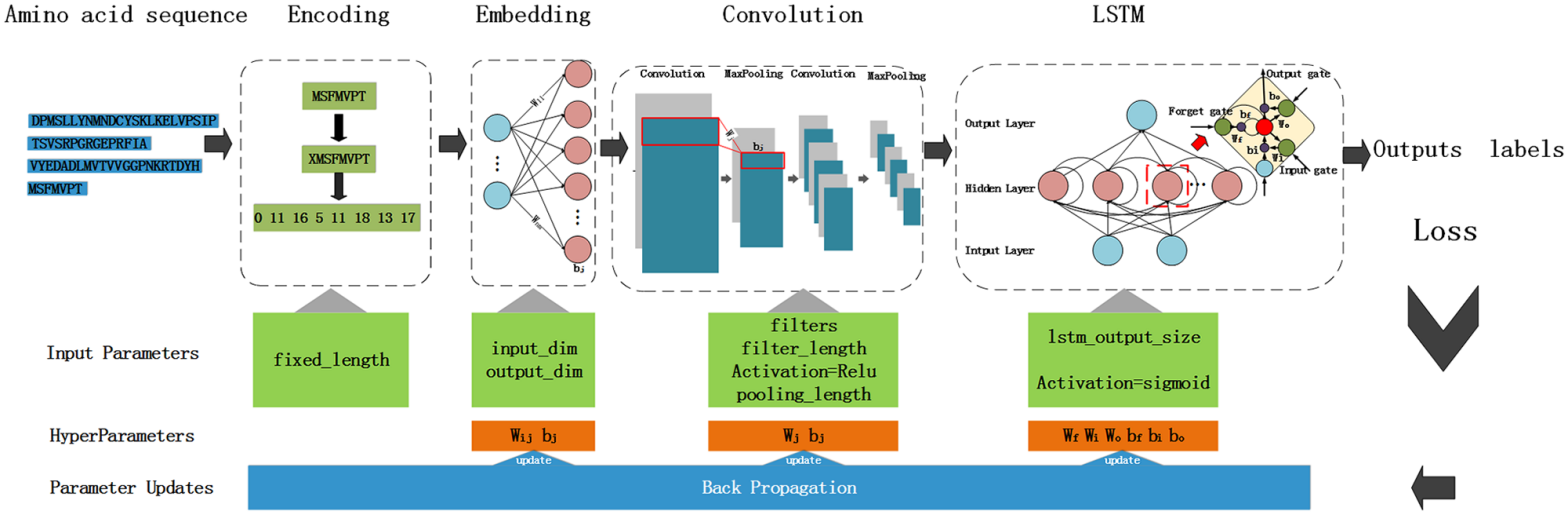
Architecture of the deep learning model.

The proposed deep learning model consists of four layered components: an encoding layer, an embedding layer, a CNN layer and a LSTM layer, shown in Fig 1. The encoding layer maps a sequence to a fixed length digital vector. The embedding layer translates it into a continuous vector. Similar to the word2vec model, transforming into this continuous space allows us to use continuous metric notions of similarity to evaluate the semantic quality of individual amino acid. The CNN layer consists of two convolutional layers, each followed by a max pooling operation. The CNN can enforce a local connectivity pattern between neurons of layers to exploit spatially local structures. Specifically, the CNN layer is used to capture non-linear features of protein sequences, e.g. motifs, and enhances high-level associations with DNA binding functions. The Long Short-Term Memory (LSTM) networks capable of learning order dependence in sequence prediction problems are used to learn long-term dependencies between motifs.

A given protein sequence *S*, after four layer processing, an affinity score *f*(*s*) to be a DNA-binding protein is calculated by the Eq 1.

$$\begin{array}{c} \begin{array}{c} f(s)=LSTM(CNN(Embedding(encoding(s))) \end{array} \end{array}$$ (1)

After that, a sigmoid activation is applied to predict the function label of a protein sequence and an binary cross-entropy is applied to assess the quality of networks. The whole process is trained in the back propagation fashion. Fig 1 shows the details of the model. To illustrate how the proposed method works, an example sequence *S* = *MSFMVPT* is used to show products after each processing.

#### Protein sequence encoding

Feature encoding is a tedious but critical work for building a statistical machine learning model in most of protein sequence classification tasks. Various approaches, such as homology-based methods, n-gram methods, and physiochemical properties based extraction methods, etc, have been proposed. Although those methods work well in most scenarios, human intensive involvement lead to less useful practically. One of the most success in the emerging deep learning technology is its capability in learning features automatically. In order to verify its generality, we just assign each amino acid a nature number, see Table 5. It should be noted that the orders of amino acids have no effects on the final performance.

**Table 5:** The amino acids encoder.

Amino acids	Letters	Code
Alanine	A	1
Cysteine	C	2
Aspartic	D	3
Glutamic	E	4
Phenylalanine	F	5
Glycine	G	6
Histidine	H	7
Isoleucine	I	8
Lysine	K	9
Leucine	L	10
Methionine	M	11
Asparagine	N	12
Proline	P	13
Glutamine	Q	14
Arginine	R	15
Serine	S	16
Threonine	T	17
Valine	V	18
Tryptophan	W	19
Tyrosine	Y	20
Illegal Amino acids	B, J, O, U, X, Z	0

The encoding stage just generates a fixed length digital vector of a protein sequence. If its length is less than the “*max_length*”, a special token “X” is filled in the front. As the example sequence, it becomes 2 after the encoding.

$$\begin{array}{c} \begin{array}{c} S_{1}=encoding\left(S\right)=\left(0,11,16,5,11,18,13,17\right) \end{array} \end{array}$$ (2)

#### Embedding stage

The vector space model is used to represent words in natural language processing. Embedding is a map process that each word in the discrete vocabulary will be embed into a continuous vector space. In this way, Semantically similar words are mapped to similar regions. This is done by simply multiplying the one-hot vector from left with a weight matrix *W* ∈ *R*^*d* × |*V*|^, where |*V*| is the number of unique symbols in a vocabulary, as in (3).

$$\begin{array}{cc} e_{t} & =Wx_{t} \end{array}$$ (3)

After the embedding layer, the input amino acid sequence becomes a sequence of dense real-valued vectors (*e*_1_, *e*_2_, …*e*_*t*_). Existing deep learning development toolkits Keras provide the embedding layer that can transform a (*n*_*batches*, *sentence*_*length*) dimensional matrix of integers representing each word in the vocabulary to a (*n*_*batches*, *sentence*_*length*, *n*_*embedding*_*dims*) dimensional matrix. Assumed that the output length is 8, The embedding stage maps each number in *S*_1_ to a fixed length of vector. *S*_1_ becomes a 8 × 8 matrix (in 4) after the embedding stage. From this matrix, we may represent Methionine with [0.4, −0.4, 0.5, 0.6, 0.2, −0.1, −0.3, 0.2] and represent Thyronine with [0.5, −0.8, 0.7, 0.4, 0.3, −0.5, −0.7, 0.8].

$$\begin{array}{c} S_{2}=(\begin{array}{cccccccc} 0.1 & -0.4 & 0.1 & 0.2 & 0.6 & 0.4 & -0.1 & 0.1\\ 0.4 & -0.4 & 0.5 & 0.6 & 0.2 & -0.1 & -0.3 & 0.2\\ 0.2 & -0.2 & 0.6 & 0.7 & -0.1 & 0.1 & -0.2 & 0.1\\ 0.5 & -0.2 & 0.1 & 0.6 & 0.2 & -0.6 & -0.2 & 0.9\\ 0.4 & -0.4 & 0.5 & 0.6 & 0.2 & -0.1 & -0.3 & 0.2\\ 0.8 & -0.5 & 0.4 & 0.7 & 0.5 & -0.2 & -0.5 & 0.3\\ 0.9 & -0.6 & 0.7 & 0.8 & 0.2 & -0.1 & -0.2 & 0.7\\ 0.5 & -0.8 & 0.7 & 0.4 & 0.3 & -0.5 & -0.7 & 0.8 \end{array}) \end{array}$$ (4)

#### Convolution stage

Convolution neural networks are widely used in image processing by discovering local features in the image. The encoded amino acid sequence is converted into a fixed-size two-dimensional matrix as it passed through the embedding layer and can therefore be processed by convolutional neural networks like images. Let *X* with dimension *L*_*in*_ × *n* be the input of a 1D convolutional layer. We use *N* filters of size *k* × *n* to perform a sliding window operation across all bin positions, which produces an output feature map of size *N* × (*L*_*in*_ − *k* + 1). As the example sequence, the convolution stage uses multiple 2-dimension filters *W* ⊆ *R*^2×8^ to detect these matrixes, as in (5)

$$\begin{array}{cc} x_{j}^{l} & =f(x_{i}^{l-1}⊗W_{j}+b_{j}^{i}) \end{array}$$ (5)

Where *x*_*j*_ is the *j*—*th* feature map, *l* is the number of the layer, *W*_*j*_ is the *j*—*th* filter, ⊗ is convolution operator, *b* is the bias, and the activation function *f* uses ‘Relu’ aiming at increasing the nonlinear properties of the network, as shown in (6).

$$\begin{array}{cc} f(x) & =max(0,x) \end{array}$$ (6)

**Fig 2:**
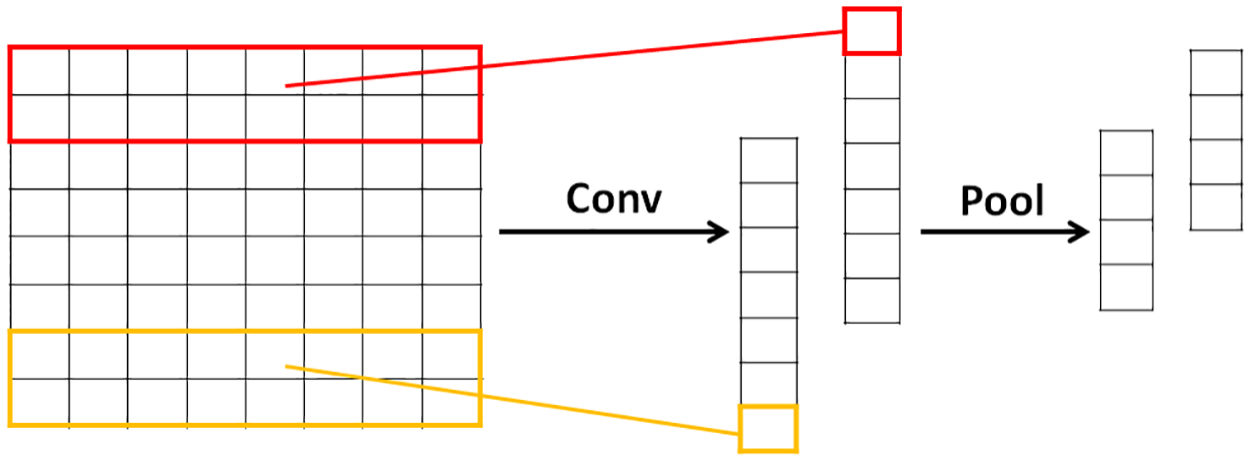
The structure of convolution neutral network. The model uses 2 filters to obtain 2 feature maps, then apply a max-overtime pooling operation over the feature map and take the maximum value as the feature corresponding to the filter.

The structure of convolution neural network is shown in Fig 2. Each filter is used to scan a feature in the sequence. In order to understand the convolution neural network more intuitive, we take out a 2 × 8 filter (7) in the convolution layer from the model trained with the best performance.

$$\begin{array}{c} W=(\begin{array}{cccccccc} 0.1 & 0.2 & 0.3 & -0.5 & 0.1 & 0.3 & -0.1 & 0.2\\ 0.2 & -0.1 & 0.2 & -0.7 & 0.1 & -0.3 & -0.2 & 0.4 \end{array}) \end{array}$$ (7)

*W* is used to detect the *S*_2_, a 8—dimension vector is obtained, as shown below.

$$\begin{array}{c} r=conv\left(S_{2}\right)=(\begin{array}{c} 0.06\\ 0\\ 0\\ 0\\ 0\\ 0\\ 0.61 \end{array}) \end{array}$$

Then, a max-overtime pooling operation with the *pooling*_*length* = 2 is applied, *r* becomes *S*_3_ (in the Eq 8), which known as a feature map detected by the filter *W*.

$$\begin{array}{c} S_{3}=pool\left(r\right)=(\begin{array}{c} 0.06\\ 0\\ 0\\ 0.61 \end{array}) \end{array}$$ (8)

#### LSTM stage

**Fig 3:**
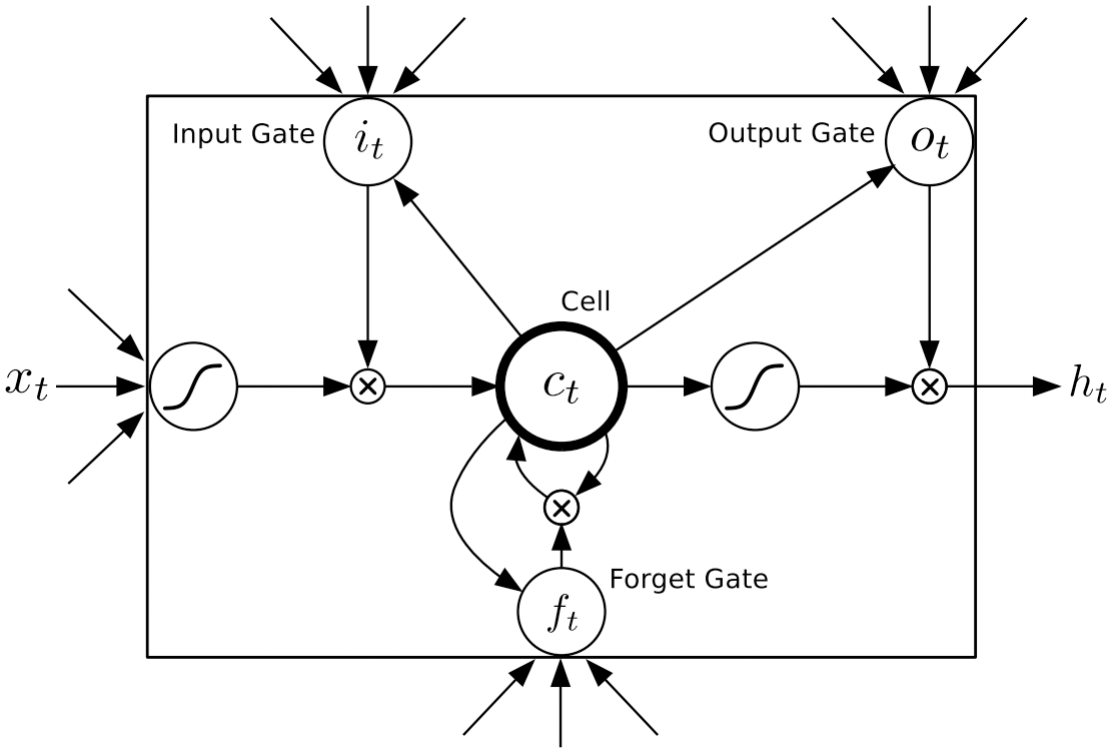
Long short-term memory cell.

Although traditional RNNs have achieved significant results in speech recognition and text generation, the problem of vanishing and exploding gradients has made it difficult to learn long-term dynamics. LSTM is a special recurrent neural network architecture and provides a solution by incorporating memory units that allow the network to learn when to forget previous hidden states and when to update hidden states given new information. It uses purpose-built memory cells to store information. The classical structure of a LSTM cell [26] is shown in Fig 3.

$$\begin{array}{ccc} & i_{t}= & \sigma (W_{xi}x_{t}+W_{hi}h_{t-1}+W_{ci}c_{t-1}+b_{i}) \end{array}$$ (9)

$$\begin{array}{ccc} & f_{t}= & \sigma (W_{xf}x_{t}+W_{hf}h_{t-1}+W_{cf}c_{t-1}+b_{f}) \end{array}$$ (10)

$$\begin{array}{ccc} & c_{t}= & f_{t}c_{t-1}+i_{t}tanh\left(W_{xc}x_{t}+W_{hc}h_{t-1}+b_{c}\right) \end{array}$$ (11)

$$\begin{array}{ccc} & o_{t}= & \sigma (W_{xo}x_{t}+W_{ho}h_{t-1}+W_{co}c_{t}+b_{o}) \end{array}$$ (12)

$$\begin{array}{ccc} & h_{t}= & o_{t}tanh\left(c_{t}\right) \end{array}$$ (13)

Components of a LSTM cell is explained by equations above. where *σ* is the logistic sigmoid function, and i, f, o and c are respectively the input gate, forget gate, output gate, cell and cell input activation vectors, all of which are the same size as the hidden vector h. The weight matrix subscripts have the obvious meaning, for example *W*_*hi*_ is the hidden-input gate matrix, *W*_*xo*_ is the input-output gate matrix etc. The weight matrices from the cell to gate vectors (e.g. *W*_*ci*_) are diagonal, so element m in each gate vector only receives input from element m of the cell vector. The bias terms (which are added to i, f, c and o) have been omitted for clarity.

In our model, the features from previous stage are passed to LSTM network as input. The LSTM generates fixed length feature representation of the output represented by *S*_4_ from the second convolution layer.

$$\begin{array}{c} \begin{array}{c} S_{4}=LSTM\left(S_{3}\right)=\left(a_{1},a_{2},\ldots ,a_{n}\right) \end{array} \end{array}$$

#### Activate and loss functions

In general, a sigmoid function demonstrates well mathematics behaviors such as real-valued, differentiable, having a non-negative or non-positive first derivative, one local minimum, and one local maximum. So, in this work, we use it as the activation function of the network, see Eq 14.

$$\begin{array}{c} \begin{array}{c} o=sigmoid\left(S_{4}\right)=1/\left(1+e^{-S_{4}}\right) \end{array} \end{array}$$ (14)

A loss function measures how well a machine learning model fits empirical data. In this study, a binary cross entropy [27]. is applied to assess the prediction performances, see Eq 15.

$$\begin{array}{c} \begin{array}{c} binary\_crossentropy(t,o)=-(t(log(o)+(1-t)log(1-o)) \end{array} \end{array}$$ (15)

Where *t* is the target and *o* is the output.

The whole process is implemented in Keras framework, a minimalist and highly modular neural networks library. Keras is written in Python and capable of running on top of either TensorFlow or Theano. It was developed with a focus on enabling fast experimentation, and supported both CPU and GPU.

## Results

### Experiment setups

We used three kinds of datasets including balanced, unbalanced and multi-species to benchmark the performance of different models. For each dataset, 80%of them are chosen randomly for training, the rest of them for testing. The final performance is given via the best of the k-fold (*k* = 3, 5, 10) cross validations.

All the experiments use same parameters for the network. The input parameters and output sizes of each layer are shown in Table 6.

**Table 6:** The parameters and output sizes of each layer.

Layers	Parameters	Output_size
Input	sentence_length = 1000	(128, 1000)
	n_batches = 128	
Embedding Layer	input_dim = 21	(128, 1000, 128)
	output_dim = 128	
Convolution Layer 1	filters = 64	(128, 991, 64)
	filter_length = 10	
	activation = relu	
MaxPooling	pooling_length = 2	(128, 496, 64)
Convolution Layer 2	filters = 64	(128, 492, 64)
	filter_length = 5	
	activation = relu	
MaxPooling	pooling_length = 2	(128, 246, 64)
Lstm Layer	lstm_output_size = 70	(128, 70)
Output	activation = sigmoid	(128, 1)

### Evaluation measures

To evaluate the performance of the proposed method, a couple of assessment measures are used in this study. These criteria includes accuracy, sensitivity, specificity. There are defined in Eqs from 16 to 18.

$$\begin{array}{c} \begin{array}{c} Accuracy=\frac{TP+TN}{TP+TN+FP+FN}*100\% \end{array} \end{array}$$ (16)

$$\begin{array}{c} \begin{array}{c} Sensitivity=\frac{TP}{TP+FN}*100\% \end{array} \end{array}$$ (17)

$$\begin{array}{c} \begin{array}{c} Specificity=\frac{TN}{TN+FP}*100\% \end{array} \end{array}$$ (18)

Where TP, TN, FN, and FP are the numbers of true positives, true negatives, false negatives, and false positives, respectively. Among these measures, the sensitivity indicates the accuracy of predicting positive samples, the specificity indicates the accuracy of predicting negative samples, and the accuracy is defined as the ratio of correctly predicted samples in test set.

Additionally, the area under a receiver operating characteristic curve (AUC) is also applied to evaluate the performances. AUC is a robust overall measure because its calculation relies on the complete ROC curve and thus involves all possible classification thresholds.

### The results in equal data set

**Fig 4:**
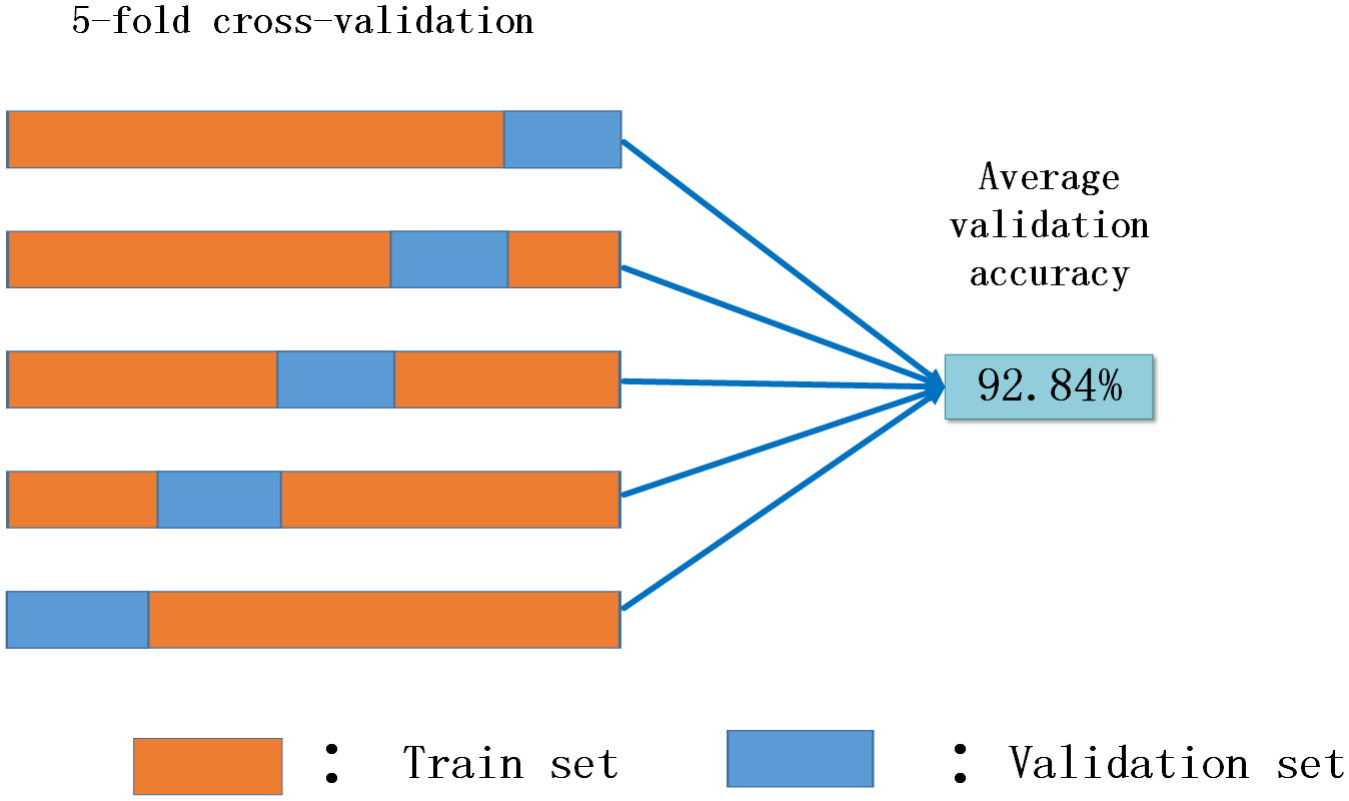
Results of 5-fold cross validation.

To demonstrate the ability of the proposed method for predicting DNA binding proteins, we first evaluate it on the independent testing dataset by the k-fold (*k* = 3, 5, 10) cross validation. In the k-fold cross-validation, protein sequences are randomly divided into *k* equal parts. In each experiment, one part is kept for the testing set and the other *k* − 1 parts are used as the training set. The accuracies for 3, 5 10-fold experiments are 87.5%, 92.8%and 93.1% respectively. Then we use the best model of in the 5-fold experiment (see Fig 4) to test sequences from Arabidopsis and Yeast species [25], compare the accuracies with the ones with DNA binder and LibSVM predictions, see Table 7. The results show that the prediction accuracies of our model outperform LibSVM nearly by 8% and 4% for Arabidopsis and yeast species respectively.

**Table 7:** The prediction accuracies across different models.

Model	Test data set	Accuracy
LibSVM	Arabidopsis(200)	0.81
	Yeast(200)	0.76
DNA Binder(10)	Arabidopsis(200)	0.74
	Yeast(200)	0.67
**Ours**	Arabidopsis(200)	**0.89**
	Yeast(200)	**0.80**

### The results in realistic data set

**Fig 5:**
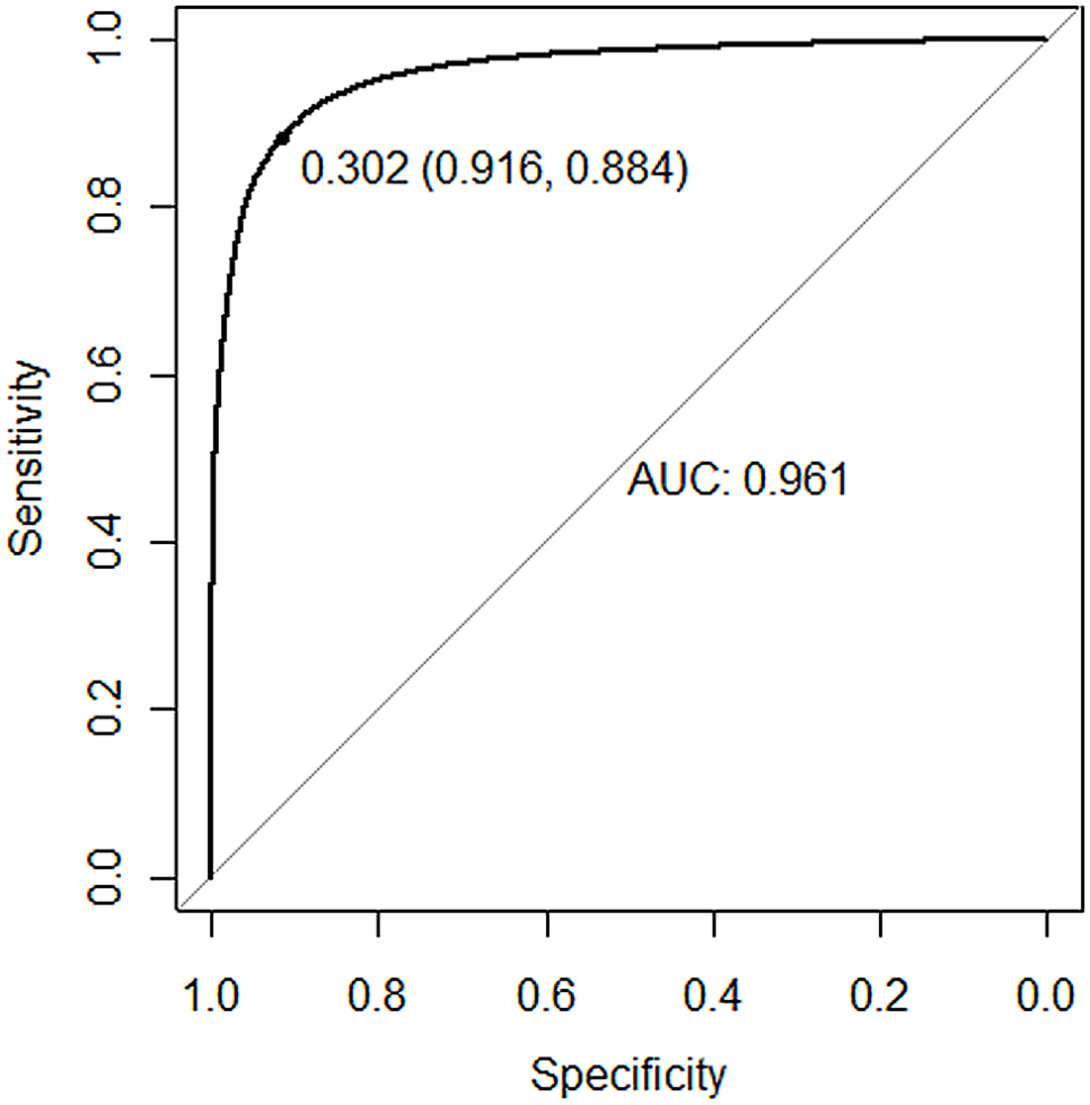
The ROC of the test set.

**Fig 6:**
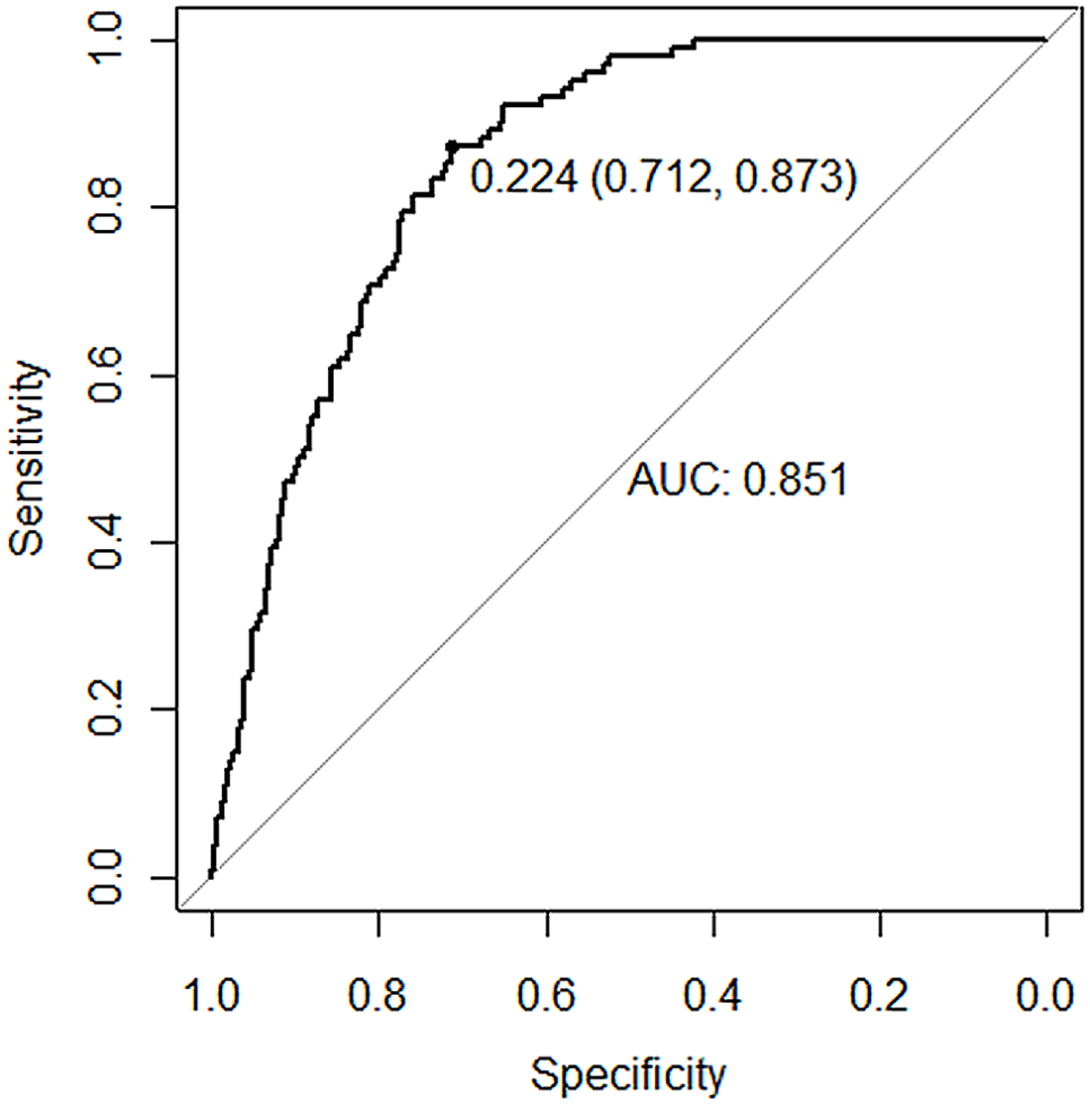
The ROC of the validation set.

For the realistic dataset, we calculate their accuracy, sensitivity, specifity and auc values shown in Table 8 and draw the ROC curves for testing and validation datasets in Figs 5 and 6 respectively.

**Table 8:** The results in the realistic data set.

Test data	Acc	Sensitivity	Specifity	Auc
Test data(76,748)	0.942	0.884	0.916	0.961
Validation data(584)	0.825	0.873	0.712	0.851

From the results, we can see that our model works well for both of class imbalanced and balanced datasets with the competitive ROC behaviors, which is a very hard situation for traditional machine learning methods.

### Results in multi-species dataset

To further verify the generality of our method across species, we train three models for human, mouse and rice species, then use these models to test others. Table 9 shows the results across different species.

**Table 9:** The results in multi-species dataset.

Train set	Test set	Accuracy
human(11,092)	human(2,772)	0.8294
	mouse(1,952)	0.839
	rice(1,800)	0.739
mouse(7,814)	human(2,772)	0.798
	mouse(1,952)	0.7473
	rice(1,800)	0.7479
rice(7,202)	human(2,772)	0.75
	mouse(1,952)	0.719
	rice(1,800)	0.918

From the results, human model works well in both itself and mouse, and vice versa. The rice model works well for itself, but lower accuracy in human and mouse. These results coincide the fact that human has close genetic relationship with mouse, is far from the rice.

### Performance comparisons with different feature extraction methods

We also compare the performances of our deep learning model with other feature extraction methods on both of the equal datasets and realistic datasets.

Three kinds of feature extraction methods, including 188D [28], Auto Covariance(AC) [29] and Conjoint triad(CT) scores [30] are used, and the linear regression, support vector machine, random forests are applied to test the performances of those features.

The 188D extracted the sequence characteristics according to the composition, distribution and physiochemical properties of amino acids and then formed a 188-dimension vector to represent the raw sequence. The AC method took into account the interactions between amino acids within the entire sequence, and represented each sequence by a vector of AC variables describing the average interactions between residues. The CT method divided all amino acids into seven categories and regarded three consecutive amino acids as a unit, and represented each sequence by a 343-dimension vector of the frequencies of triad types appearing in the amino acid sequence.

The performance comparisons using the same training and testing datasets are summarized in Tables 10 and 11 respectively. For the equal dataset, our model outperforms 2% of the best known previous results by the combination of 188D and SVM, and more than 10% by the average of all others. For the realistic dataset, our model performs similar accuracy with the best (188D+RF) of others, but outperforms its sensitivity and AUC by 0.2627, and 0.1511 respectively. This suggests that the proposed model is more reliable and robust.

**Table 10:** Performance comparisons on the equal dataset.

Method		Accuracy
188D	LR	0.7607
	SVM	0.9078
	RF	0.8776
AC	LR	0.6789
	SVM	0.7993
	RF	0.8360
CT	LR	0.7080
	SVM	0.8565
	RF	0.8588
**Our model**		**0.9284**

**Table 11:** Performance comparisons on the realistic dataset.

Method		Accuracy	Sensitivity	Specificity	Auc
188D	LR	0.8940	0.1442	0.5560	0.5651
	SVM	0.9500	0.6057	0.9029	0.7989
	RF	0.9581	0.6213	0.9801	0.8099
AC	LR	0.8922	0.0220	0.5690	0.5100
	SVM	0.9255	0.3296	0.9495	0.6637
	RF	0.8919	0.0308	0.5949	0.5141
CT	LR	0.8925	0.0898	0.5745	0.5408
	SVM	0.9219	0.3230	0.8969	0.6592
	RF	0.8920	0.0290	0.6055	0.5133
**Our model**		**0.942**	**0.884**	**0.916**	**0.961**

### Performance comparisons with low-redundancy training sets

We train the models on the low-redundancy versions of the equal and realistic datasets, and compare the performances with 188D+SVM method. The results are shown in Tables 12 and 13.

**Table 12:** The results in low-redundancy equal data set.

Method	Accuracy
full model	0.928
low-redundancy model	0.8849
188D+SVM	0.8745

**Table 13:** The results in low-redundancy realistic data set.

Method	Accuracy	Sensitivity	Specificity	Auc
full model	0.942	0.884	0.916	0.961
low-redundancy model	0.8638	0.5138	0.8461	0.7129
188D+SVM	0.8435	0.3340	0.7063	0.6502

For the low-redundancy version of equal dataset, the accuracy is lower 3.86% than full version and slight higher than 188D+SVM method. When the model is applied to Arabidopsis and yeast datasets, the accuracies are 85% and 78% respectively, which are slight lower than ones in the full model.

For the low-redundancy version of realistic dataset, its model works worse than the full model, but better than the 188D+SVM method over all the measures.

These results suggest that the sequence redundancy in the training dataset does not decrease the performances, while somehow increase the power of prediction capability because deep learning requires huge volume of data to fit its model and provides mechanisms (especially the dropout technology) to overcome model over-fitting.

## Discussion

In computer vision area, recent research [31] reveals that networks depth is of crucial importance, for example on the challenging ImageNet, models were exploited with a depth sixteen [31] to thirty [32]. In order to compare the effects of different depths of networks and lengths of filters, we have designed two other models. The first contrastive model is a single layer CNN with the filter length 5. The second one has two layers CNN with the filter length 5 of the first CNN layer.

**Fig 7:**
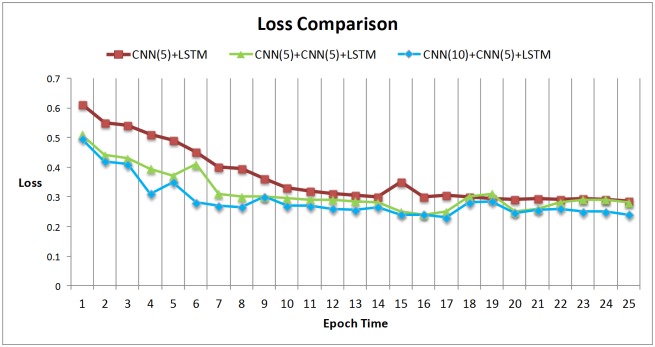
Loss comparisons in different models.

**Fig 8:**
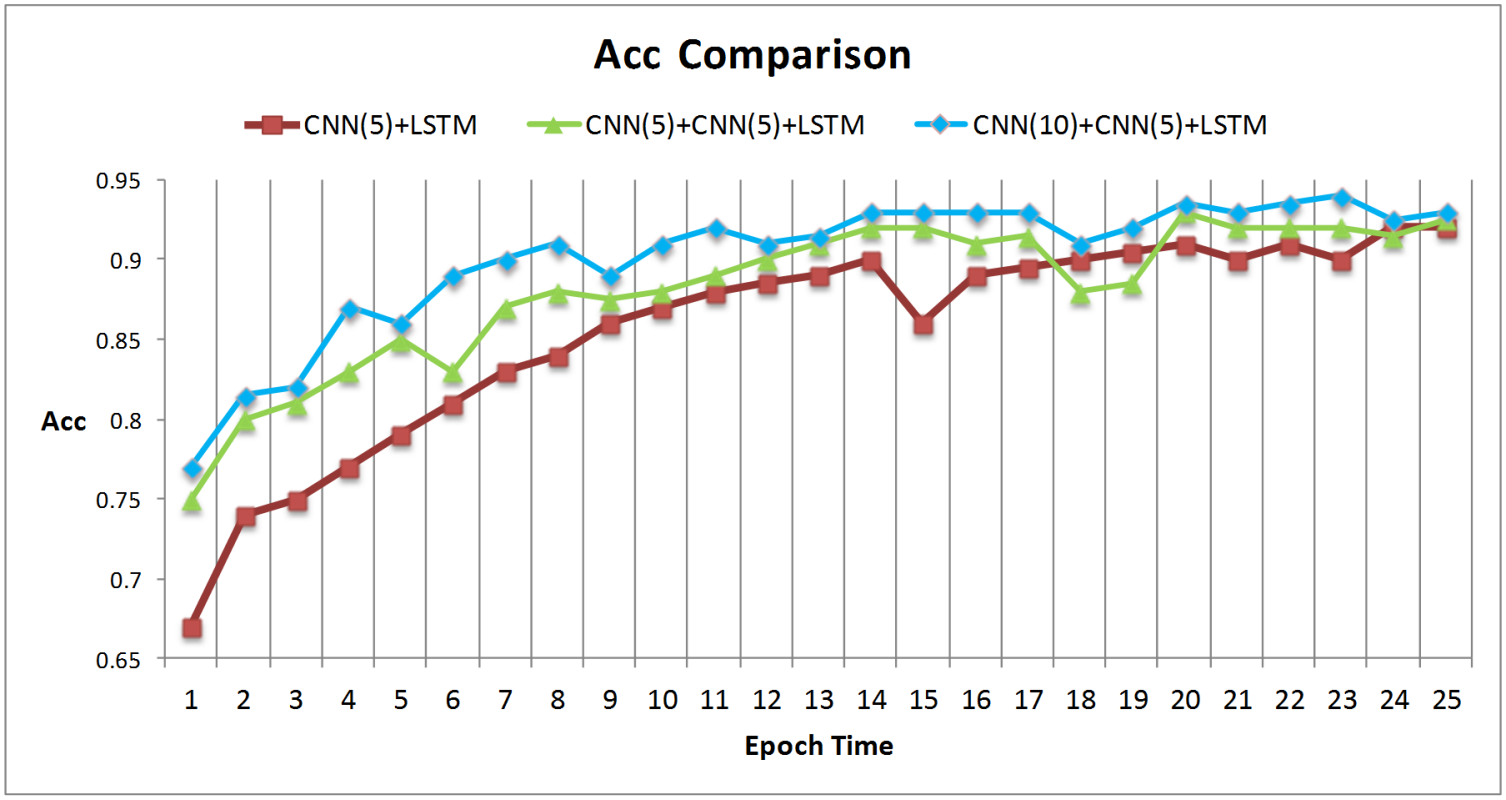
ACC comparisons in different models.

The Fig 7 shows that the model with two layers CNN can speedup the convergence of loss functions, while the model with large filter length can get higher convergence of loss functions. The prediction accuracy demonstrates similar behaviors, see Fig 8.

During the experiments, we found that the performances of the neural networks are somehow driven by data rather than the structures designed artificially. For small datasets, deep learning doesn’t have a more excellent performance than traditional machine learning methods. With the rapidly growth of protein sequence data, the advantage of deep learning can be reflected increasingly. Meanwhile the speed of computing is an indispensable problem. GPUs are often used to accelerate the computational speed for this situation.

A most recent work predicted DNA-binding proteins interacting with ssDNA (single-stranded DNA) or dsDNA (double-stranded DNA) using OAAC (overall amino acid composition) features, dipeptide compositions, PSSM (position-specific scoring matrix profiles) and split amino acid composition (SAA) [33]. Testing by SVM (support vector machine) and RF (random forest) classification model, their method can achieve the accuracy of 88.7% and AUC of 0.919. Our method achieve the accuracy of 94.2% and AUC of 0.961 on the realistic data set. Moreover, the deep learning approach can speedup the procedures of trivial feature selection and enable scientists put more efforts on biological analysis.

All the source codes used in this study are available at the figshare server (https://doi.org/10.6084/m9.figshare.5231602.v1). A user-friendly web-server for predicting DNA binding proteins is accessible at http://119.23.251.26/WebServer/.

## Conclusion

Computational biologists are often be struggling to successfully extract meaningful features and choose an appropriate machine learning algorithm in predicting spatial structures or functions of biological sequences. The deep learning framework capable of learning features automatically and training models in a back propagation way is making a big success towards these fields. In this paper, we presented a deep learning based approach for predicting DNA binding functions of proteins only using primary sequences. The two layers of CNN plus LSTM networks allow for an increase in learning power and contain more potential for motif refinements in both of local connectivity and long-term dependence.

Compared with DNA binder and LibSVM, the proposed method shows a state-of-the-art performance on both of the equal and realistic data sets. It also demonstrates substantial generality across multi-species testing. Moreover, the method outperforms most of the existing feature extraction methods plus a successful machine learning algorithm in terms of accuracy, specificity, sensitivity and AUC. This comprehensive investigation of the deep learning model in predicting DNA binding functions of proteins might yield a competitive tool for future proteomics studies. The proposed deep learning approach would have many other potential applications, such as protein remote homology detection [34], miRNA prediction [35], etc.
